# A qualitative inquiry of sexuality in Iranian couples using the Information-Motivation-Behavioral skills paradigm

**DOI:** 10.1186/s42506-019-0024-7

**Published:** 2019-12-17

**Authors:** Effat Merghati Khoei, Babak Moeini, Majid Barati, Ali Reza Soltanian, Ehsan Shahpiri, Ali Ghaleiha, Fahimeh Bagherikholenjani

**Affiliations:** 1 The Iranian National Center for Addiction Studies (INCAS), Institute of Risk Behavior Reduction, Tehran University of Medical Sciences, Tehran, Iran; 2 Family & Sexual Health Division in the Brain & Spinal Cord Injury Research Center (BASIR), Neuroscience Institution, Tehran University of Medical Sciences, Tehran, Iran; 3 Social Determinants of Health Research Center & Department of Public Health, School of Public Health, Hamadan University of Medical Sciences, Hamadan, Iran; 4 Modeling of Non communicable Diseases Research Center & Department of Biostatistics, School of Public Health, Hamadan University of Medical Sciences, Hamadan, Iran; 5 School of Educational Sciences and Psychology, Khorasgan Islamic Azad University, Isfahan, Iran; 6 Department of Psychiatry, School of Medicine, Hamadan University of Medical Sciences, Hamadan, Iran; 7 Health Education and Health Promotion, School of Public Health, Hamadan University of Medical Science, Hamadan, Iran

**Keywords:** Sexual information, Sexual motivation, Sexual behaviors, Couple, Iran

## Abstract

**Background:**

Sexuality is interwoven with individuals’ information, motivation, and behaviors.

**Objective:**

To explore sexually related information, motivation, and behaviors that Iranian newly married couples utilized through their marital lives.

**Methods:**

We employed in-depth face-to-face interviews with 22 couples between the ages of 21 and 35 years to collect rich qualitative data. Content analysis was used to analyze the data. Our data analysis process was guided by the Information-Motivation-Behavioral skills (IMB) model as a potential framework for understanding of the participants’ sexual and marital lives**.**

**Results:**

IMB’s conceptual bases were adequately reflected in the participants’ sexual narratives. The participants highlighted information needs related to their sexual relationships and services that should be provided by the relevant programs in the educational and national health system. Fulfillment of each other’s sexual needs was identified as the most important motivation of the participants. Sexual needs of husband, love, and liking were the main motivations for women’s sexual submission. The main behaviors found included couples’ communication skills and performing using feminine traits by women in order to fit the role of a sexually skillful wife.

**Conclusion:**

Our data analysis revealed that couples shared a proper comprehension of each other’s means of sexual behaviors. A dominance of religious discourse, non-verbal, mostly physical means of communication was employed by the couples to express or initiate sexual interactions. Furthermore, our findings support the utility of IMB as a potential basis for understanding married couples’ sexual lives. Our data highlight an implication to expand the motivation structure of the IMB model to incorporate an individual’s sexual understandings and the sexual needs to promote mutual and pleasurable sexual life within the Iranian culture.

## Introduction

Sexuality is a significant aspect of human life that plays an important role in one’s health and well-being. According to the WHO definition, sexuality is experienced and expressed in thoughts, beliefs, attitudes, values, desires, fantasies, behaviors, practices, roles, and relationships [1]. Despite recognizing biological effects on sexuality, scientists generally believe that sexuality is a social phenomenon based on defined arrangements that provide guidelines for appropriate sexual behavior [2]. Sexual behavior is the product of a deeper and more complex process called sexual socialization [3], which includes the development of sexual identity and sexual role, the acquisition of information and skills, and finally the formation of sexual attitudes [4]. The family and community are two main institutions that influence how people handle this process [5]. A basic right of the community members is to provide them with facilities and tools to understand their sexual responsibility and equip them with sexual information and skills [6]. Having proper sexual information and skills helps couples experience more enjoyable and responsible sexual life [7].

Research in the area of sexual health in Iran shows that the level of sexual dysfunction is high in Iranian women and men [8, 9]. According to the results of a systematic review in 2015, the prevalence of sexual disorders in Iran was 35.2% [10]. The divorce rate has also risen in recent years [5] and one of the effective factors in these divorces is the low level of satisfaction of couples with their sexual lives [11]. So, equipping individuals, couples, families, and society with the sexual information, motivation, and behavioral skills appropriate to age, gender, religion, race, socioeconomic context, physical and cognitive abilities, and cultural values and norms is the best way to ensure the healthy and satisfying sexual behaviors and limiting the risks caused by inappropriate sexual behaviors in young people [12, 13].

The Information-Motivation-Behavioral skills (IMB) model was used as the main theoretical framework of this study, regarding the determinant role of information, motivation, and behavioral skills in promoting sexual behaviors and subsequent sexual health. Several studies have shown the effectiveness of this model as a strong theoretical framework for sexual health [14–16]. The IMB model includes three primary constructs that influence sexual behavior changes. Information includes three parts of the facts, heuristics, and implicit theories. Facts are couples’ correct information about sexual relationships that serve as a guide to promoting their sexual welfare. Heuristics refers to applying couples’ experiences as an aid to learn, discover, or solve sexual problems by experiential methods, and in particular tests and errors. Implicit theories are personal structures in the context of sexual relationships which is in the minds of the couples. Motivation includes both personal and social motivations. Personal motivation is positive or negative attitudes toward sexual relationships. Social motivation includes individual beliefs in relation to social norms or couple’s perceptions of social support associated with sexual health behaviors. Acquiring appropriate sexual skills is necessary to change the sexual behavior of couples. Behavioral skills include practical skills that are the objective abilities of couples (for example couples know how to negotiate together) and self-efficacy or couple’s belief in their ability to implement preventive behaviors for sexual problems or sexual health promotion behaviors (couples’ beliefs about their ability to successfully negotiate) [17].

Sexual concepts are subjective and context-based concepts in which social-cultural relationships, as well as the value framework and beliefs governing any society, dominate this concept. In such a way, this concept must be considered in each society in relation to its particular context [4]. This attribute attests to the parallelism of this concept with the worldview of the qualitative method. The purpose of this study was to identify sexual information, motivation, and behavioral skills of Iranian couples in Isfahan, Iran.

## Methods

The reported data were collected as the first phase of a PhD research project exploring the Iranian couples’ understanding of sexuality. IMB model constructions were inspired as the theoretical basis for the development and implementation of the study.

### Population

Participants were new couples. New couples refer to people who had been married within the past 5 years [18]. A total of 22 couples (*n* = 22 women; *n* = 21 men) participated in the study. The reason there was one less male participant was due to his non-participation while his wife participated. Approaching couples was conducted through an invitation entitled “Enriching marital relationships of new couples” that invited new couples to share [their] views for a study of sexual relationships. Advertisements of this invitation were placed on social networking sites and in online classifieds (e.g., Telegram, Instagram, and Linked In). Seventy-two potential people responded to the invitation and sent their contact detail to be contacted. The research team announced the research aim through the accessible social network and then contacted the volunteers and screened them for eligibility.

The inclusion criteria were (1) got married within the past 5 years, (2) no diagnosed mental illness, (3) no self-reported marital conflict, (4) residing in Isfahan city, Iran, (5) willingness to participate, and 6) willingness to share their sexual life experiences with the research team.

The assessment of mental health problem or marital conflict was based on a thorough history taking by the main researchers (FB and EMK) as well as self-reporting at the time of appointment for the interview because concealment of these two problems is a common behavior in the Iranian culture which unless verified would represent a limitation in this study.

Couples’ ages ranged between 21 and 35 years and belonged to different socioeconomic strata, educational levels, and occupational backgrounds which are reported in Table 1.

**Table 1:** Demographic characteristics of participants (*n* = 43)

Demographic characteristics	
Mean age (years)	27.9 ± 3.4
Average length of marriage (years)	3.2 ± 1.6
Average number of offspring	0/4 ± 0.68
Education	
Bachelor	55%
Master	25%
Diploma	20%
Job status	
Self-employed	35%
Employed	25%
Housewife	25%
Student	15%
How to marry with spouse	
Traditional matchmaking	40%
Family marriage	35%
Friendship	25%

### Study design

The main data collection technique was in-depth, face-to-face semi-structured interviews. Semi-structured interviews allow for a better understanding of the participant’s perspectives on sensitive subjects such as sexual interactions. In addition, it allows participants to think more critically about the subject matter [19]. Data were collected from April to July 2017. The study was approved by the Ethics Committee of Hamadan University of Medical Sciences and agreed upon by administrators at the research sites (Isfahan University of Medical Sciences). Due to cultural sensitivities, interviews with female and male participants were conducted by interviewers of the same sex. The interviews with a couple were conducted simultaneously in separate rooms at a psychological counseling clinic located in the downtown Isfahan city, where couples from different parts of the city could easily attend in interviewing sessions.

The first author contacted the eligible couples and coordinated the time and place of interviews with them. At the beginning of each interview session, a detailed explanation about research aim was presented and the participants were assured of confidentiality. At the same time, the participants were given the opportunity to ask questions and affirm their right to withdraw from the study at any time. Verbal and written informed consent were also obtained. All the couples were interviewed once and only one man refused to participate in the study.

Interviews with women were carried out by the first investigator (FB) who is a doctoral candidate of health education and health promotion and employee of Isfahan Health Center. Interviews with men were done by the fifth author (ESH) who is a doctoral candidate of consultation and works in the field of couples’ marital relationships. Both interviewers were from Isfahan and were largely familiar with the culture of participating couples. They had enough experience and skills in conducting the qualitative interviews and were also trained for sexual interview by the first author (EMK) who is a sexologist.

The main research question was “what is the status of information, motivation, and behavioral skills of Iranian couples in the field of sexuality?” Each interview began with a general and more well-known question about the participants’ life history because in Iranian culture, it is not easy to talk about sexuality and often associated with shame. Then the interviewer gradually moved to those aspects more directly related to the interview guide such as how do you describe sexual relations and their formation? Probing questions were employed according to the participant’s dialogs, to extract more details and to clarify the interviewees’ explanations, for example, “Can you tell me more about that?” [20].

Each interview lasted between 50 and 70 min and was carried out in the Persian language. Note-taking within the interview was used for managing the interview and data analysis. Interviews continued until data saturation was attained, so in the three last interviews, no new information was achieved. The sessions were facilitated by using a semi-structured inventory based on the theoretical framework of the study (IMB model) (Table 2).

**Table 2:** Interview guide

1	How do you describe sexual relations and the formation?
2	How much information did you have about sexual matters during your marriage? And how much information do you have now?
3	What was your information source for sexual issues?
4	How was your attitude toward sex before and after your marriage?
5	What is your most important motivation of sexual relationships?
6	Which social norms have affected your sexual life?
7	Who are helping you to promote your sexual life or resolve your sexual problems?
8	What behavioral skills do you have for managing or improving your sexual life?

### Data management and analysis

All interviews were digitally audio recorded and immediately transcribed verbatim and then imported into the MAXQDA-12 software. The data analysis process was done concurrently and continuously with data collection based on Graneheim and Lundman’s approach [21]. Accordingly, each transcribed text was reviewed several times to achieve a total comprehension of their content. Then the transcribed text was broken down into meaningful units. Each meaningful unit was abstracted to a “condensed meaningful unit.” Codes were extracted based on these units. The two first interviews with couples were encoded independently by EMK and FB. Then, to recognize similarities and differences of codes, we compared all transcript phrases. The authors discussed any disagreements until they reached a consensus. Coding of the subsequent interviews was conducted similarly to the first.

## Results

Study participants (*n* = 43) consisted of 22 women with their husbands (21 men) and their specifications are presented in Table 1. The findings reflect the information, motivation, and behavioral skills of Iranian couples in the field of sexuality. The following general themes were derived from the interview data.

### Information

The information construct in the IMB model is a basic background for the promotion of healthy sexual behaviors in couples.

Due to the predominant discourse of religion and the sexually conservative culture in Iran, sexual interactions are permitted exclusively within the family context and after marriage. In particular, girls are taught to have their first sexual experience with their future husband. N, 32 years old and a doctoral student, who had been married for 5 years said

*"Because I grew up in a religious and traditional family, I saw sex only in the form of marriage. It was not that these relations existed outside of marriage. I myself adhered to it as my religion says it".*

The culture of silence and the value of hijab and modesty, especially for women in Iranian culture, and lack of openness toward matters of sexuality, especially before marriage, make couples unlikely to neither seek any kind of sexual information nor be recognized as sexual beings in the society. Sh, 30 years old, master’s degree educated housewife who had been married for 1 year said she has never been looking for this information.

*"I had no information before marriage; even I had not seen the male genitalia. There was a halo in my mind. Of course, I myself did not like to follow this information for a series of issues in society like Hijab and modesty and these things. There was a curtain between us and sex"*.

In the same vein, her husband (P, 28 years old, bachelor of chemistry, an educated man working in the non-governmental sector) expressed concern about the inactive role of responsible organizations of society for providing accurate and timely information to people.

*"I have a concern for a long time, why sexuality shouldn’t be opened stealthy (behaving in a cautious and surreptitious manner)? I want to know that for me and when I was a teenager, why should not the organization that has an authority to train and talk about these things? Now, I do not say fully but start at school, for example, to tell these things. From childhood, these issues begin, and as one gets older, the problem gets clearer."*

Despite the role of institutions such as families, schools, universities, health centers, and media in providing valid information on sexual issues, but all participants pointed to the inactive role of these institutions and their inefficiencies in providing relevant and timely sexual information and services.

In the expression of contributing couples, parents’ passivity in children’s sexual upbringing was characterized by silence and failure to address sexual issues, the absence of sex education at the age of adolescence and even at the time of marriage. S, 21-year-old undergraduate student, who had been married for 1 year, said that her mother had not talked about sex even during her marriage and wedding.

*"Of course, this is a tradition in our family, for example, even when a girl wants to be a bride, someone else, for example, her aunt, is talking to her in this area, her mother does not go to talk".*

Her husband (SH, 26-year-old man, food industry engineer and an employee) has a similar view in this regard:

*"In our family, like most Iranian families, there was no talk about sexual issues. That is, both my parents and me were embarrassed to talk about these issues. Maybe we were afraid that our respect would be ruined".*

Social learning theory and script theory suggest that people create and reserve “scripts” through observation of attractive and relevant models in both real life and the media that guide social behavior. Despite the lack of direct sexual education of parents to their children, participant couples said that they had modeled parents’ behaviors in the family context for their marital and sexual life indirectly in the field of mutual respect, expressing love and marital intimacy, maintaining privacy for themselves, and flexibility in their interrelationships. S, 26 years old, master of law, and housewife, pointed to this subject:

*"I say that we did not receive any training or did not learn anything from family, but in the course of everyday life, I saw the life of my parents, I saw my aunt and saw my close relatives. I learned something from them. My parents, for example, always had privacy for themselves. Their room was surely separate and they devoted time to staying at home together or go out together".*

Also, S, 25 years old, who works as a counselor in the care center of idle daughters, considers her flexibility in marital and sexual life as a result of her family’s upbringing.

*"The flexibility I learned in marriage has come from my family. For example, I'll be having sex with my husband one night, even though I do not want to have sex for that night, it comes from the same flexibility that I learned in my family. Perhaps we do not receive directly education from the family, but family-based upbringing will affect our sexual life indirectly".*

Many participants referred to the use of informal methods such as talking to friends and relatives, the experience of peers, jokes and metaphors, watching videos, social networking, and non-native media to get information. In fact, people filled out the deficiencies in formal education using these resources and mentioned it as the only way to meet their sexual needs. The major disadvantage of these methods is the mixing of accurate information with incorrect information and creating false beliefs and attitudes about sexual relationships in individuals that may harm their sexual life. H, 29 years old, an electrical engineer, who had been married for 1 year, pointed out the disadvantages of watching porn videos to gain information and skill:

*"Unfortunately, due to the false culture that exists in the community, lack of sex education, the minds of teenagers and youth go to pornography and watch porn videos. After marrying, people realize that perhaps one percent of it can be implemented, but it does not work".*

His wife, R, 26 years old, computer engineer and employee, referred to the formation of misconceptions in herself as a result of inaccurate information:

*"At the university, when I talked to my friends, I realized something about sex. Sometimes I saw the movie in dormitory. I thought, in terms of what I had heard from my friends and what I had seen in the movies that men get more pleasure in sex, and women were more likely to be harassed. It was the same in early marriage, because I had too much pain. I thought that sex is the same as women are a means to enjoy men. But after a few months my pain got better and my husband and I learned how to act in sex, I found that sexual pleasure is really two-way and could even be more for women".*

Although married couples did not have much information before marriage, the marriage was a learning process for them, so that experience was the main source of information after marriage. During the marriage process, two people get to know each other well. This recognition helps them to improve their sexual life. M, 27 years old, bachelor of literature and housewife, referred to the importance of recognizing the needs of each other.

*"After a while when couples get acquainted, a series of issues will be resolved spontaneously. For example, after some time, my husband and I learned that if we are bad, how we can make each other better, or what we like and what we do not like and this helped us to get better our marital relationships".*

Similarly, her husband (A, 32 years old and a lawyer) points to the process of learning sex after marriage in this way:

*"I did not know about sexual matters before marriage. Sex in early marriage, I was like an awkward driver who did not know how to drive, but I've found a lot of things with my wife. I think the great tools that affect sexual relationships are science and affection. The science of knowing what you should do, what you want and doing it with affection".*

### Motivation

Sexual motivation is a psychological construct that characterizes the driving force for sexual activity. Biological, social, and cultural factors affect sexual motivation [22]. The motivation construct in the IMB model includes the personal and interpersonal attitudes of couples to sexual relations, the sexual norms of society in the field of sexual relations, and the support of the family, friends, and community of the couples in order to improve their sexual life.

#### Sexual attitude

As mentioned in the previous section, couples did not have a proper attitude toward sexual relationships before marriage because of incomplete information, particularly women socialization prohibits them to be identified as a sexual being but looking for sex experience after marriage, their attitude changed and improved. M, a 34-year-old woman and beautician, said about her attitude change toward sexual matters before and after marriage:

*"I did not think about sexual matters at all, that is, I did not even like to talk about it. If my friends talked about sexual issues, I would have left the group, but when I got married and experienced this relationship, I really saw this relationship as a need for both the woman and man that was very pleasing. Now that four years have passed since our marriage, every day, I find out more about the importance of this relationship in strengthening our marital life".*

All couples had a positive attitude toward sexual interactions and referred to various motivations for sexual relationships. One of the main motivations of couples for sexual relationship was attention to sexual need. S, 27 years old, a master of industrial engineering woman who had been married for 4 years, considers that the motivation of herself and her husband for the sexual relationship is meeting the sexual need of each other and satisfying it through mutual love and interest.

*"Our motivation is the sexual need. At times, we really see that we need it. I also want to keep him satisfied, and as soon as he addresses my need, we really want to make each other happy".*

Her husband (K, 34 years old, bachelor of accounting and an employee) considers meeting sexual need as one of the most important motivations of sexual relations for couples.

*"My motivation of sexual relationships is meeting a need, an intrinsic need that both the man and the woman must respond to it. I think 80% of people, especially men, marry to meet sexual need".*

The most important motivation of all participants for having sex was love and affection to their husband/wife and getting closer through this relationship and increasing marital intimacy as a result. Z, 29 years old, bachelor of biology and a housewife, said in this regard:

*"My husband and I have sex because of the love and affection that exists between us. It's a romantic game that is a pleasure of our life, enjoyment in a hug that my husband is trying to make me rejoicing. This intimacy brings us closer together".*

Her husband’s motivation for sex was also expressing love and affection to his wife:

*"I love my wife. My motivation of sexual relations shows my love and affection to her. When sex is based on love and affection, it leads to greater understanding, serenity and intimacy between couples. (F, 30 years old, diploma, mechanic)"*

Some married women have sex to relieve themselves and their husbands of discomfort, tension, and fatigue and calm down. E, 24 years old and a self-employed woman who had been married for 5 years, used sex as a tool for relieving tension.

*"When we crush together, one way to reconcile is to have sex. Now it’s either a suggestion or doing it, and really it happens. After sex, all the problems that exist disappear and calm down".*

Most married women find sex as their duty and their motivation for establishing a relationship is obeying their husband. Sexual submission is considered a religious duty. It is also obligatory since they have signed the marital contract. M, a 26-year-old housewife, regards the acceptance of her husband’s sexual approach as the duty of the woman.

*"Behold! This is something in our religion. It is the duty of the woman to obey. Because our religion teaches that we have to obey. If your husband asks you for sex, you must accept. If you do not want to, you should not marry".*

A limited number of women have sex in order to keep their husband from extramarital relationships. They believe that men cannot control their sexual need and failure to meet their needs by their wives makes them seek these relationships outside marriage. Therefore, women are required to meet the sexual needs of their husbands in order to prevent them from extramarital relationships. A 29-year-old woman and autism coach despite confidence in her husband expressed her fears as follows:

*"Well, “Adam” also fears of the stance of the society. Because you know that every moment ladies are ready to meet the needs of my husband. Of course, he's not like that, but it's always in my mind. If I do not respond to my husband's sexual need, he may be attracted to other women to meet his needs".*

#### Sexual norms of society

Most cultures have social norms regarding sexuality. Social norms are structural factors that shape individuals’ perceptions of appropriate sexual behaviors for men and women [23]. Cultural scenarios are social determinants of sexual behaviors that recognize which behaviors are normal in any context and which ones are not [24]. The couples referred to the sexual norms of the Iranian community, including taboos surrounding sexuality, the prohibition of premarital sexual relations, the importance of preserving virginity for women, and sexual submission as factors affecting their sexual socialization.

##### Sexual issues are taboo

In Iran, as one of the traditional religious communities, the sex-based approach does not set a clear path to the sexual socialization of individuals, and sexuality is always in a state of ambiguity. Talking about sexual issues is a taboo. B, 29-year-old woman, bachelor’s degree in Persian literature and a housewife, stated if a woman has much to ask and answer in this regard, she is viewed differently and her modesty and chastity are questioned.

*"You see, our country as it is, it did not give us any information. They all said from childhood that it was so bad, and the one who was talking about these things was a dirty person".*

##### Prohibition of premarital sexual relations

Premarital sexual relationships are not religiously and socially acceptable in the Iranian culture and are considered unlawful and taboo. Most participants had a negative attitude toward sexual experience before marriage and considered it a temporary relationship to satisfy sexual needs. From a man’s viewpoint that was 27 years old, aerospace masters, having various sexual experiences before marriage leads a man to have various sexual interests. These varieties make him compare the current partner with formers, which weakens the foundations of marital life.

*"I think that at least in our society, if there is sex before marriage, it will certainly affect the relationship after marriage, now I have no doubt about sin, paradise, and hell, but the various sexual interests of men makes it reopened again after marriage if they have experienced various relationships before marrying, and this is one reason for divorce".*

In this regard, his wife (Y, 27-year-old woman, master’s degree in entrepreneurship management) stated that religious beliefs were an important factor for her in avoiding premarital sexual experience:

*"I myself tried, at least for the sake of avoiding to be guilty, not to go to these issues. Premarital sexual relations, homosexuality and masturbating, are very ugly things from God’s eyes. Perhaps if I was born in another country and I was not a Muslim, I would easily go to these things, but when they said that from God's sake, that’s not the right thing, hence I did not go to this relationship, that’s the only reason".*

##### The importance of preserving virginity for women

In Iranian culture, women’s sexual practices are organized based on shame, chastity, and honor, and emphasis is placed on preserving a women’s virginity. So, engaging in premarital sexual relationships and losing the symbol of virginity—the hymen—can cause the disrepute of a girl and her family and jeopardize her marital status and future. I, a 26-year-old man and marketer, said in this regard:

*"If women have sex before marriage, their marriage process would be very difficult, and they will have difficulty to finding the right husband and if their husband finds it out after marriage then it’s too bad".*

His wife, N, 22-year-old, student of handicrafts, said in this regard:

*"Always we are told that after the first sexual intercourse you should bleed. Your curtain will be ripped off. If you do not get blood, you are not a good girl 100% and this means that you had sex before marriage. These issues were very important in the society and are still there".*

##### Women’ sexual submission

Women’ sexual submission is one of the items of marital contract that is clearly described in the original Islamic sources such as the Quran. Almost all participating women believe that they should submit to their husbands’ sexual needs. T, 26 years old, master’s degree in medicinal plants and housewife, pointed to the necessity of sexual submission to her husband:

*"When you get married, you have to obey, and whenever your husband asks you for sex, respond to his need".*

Her husband (J, 29 years old, agricultural engineer) said in this regard:

*"Some of my elder colleagues do not even know that the woman enjoys sexual relationship too. They use her as a tool and say it is her duty to do this. I also have the duty to work and pay for her. Their women also think so. They say my husband works and gives me and kids money, so I have to do sex. Of course, the new generation does not think so".*

#### Social support

Couples’ understanding of social support will affect their sexual health behaviors. The existence of individuals or community support systems that can help couples to resolve sexual problems or promote their sexual health is important. According to the narratives of participating couples, parental conservatism in the sexual affairs of children in Iran has prevented couples from asking for parental help to resolve their sexual problems. However, couples have pointed to the use of other family members’ help in this regard. For example, S, a 25-year-old woman who was a psychology counselor, uses her sister’s help.

*"Yeah, I talk about my sexual problems with my sister, who looks very similar in spirit and appearance to me, but not with my mother at all".*

The difference in sexual expectations and sexual style of new couples with their parents was one of the obstacles to seeking help from them. B, 23-year-old woman, an employee of a tourism institution who has been married for 2 years, said:

*"Our sexual relationship is very different from our parents. For example, my husband and I, in some nights, see films together up to half the night. Of course, not a porn movie, romantic movies that may have sexy scenes. It’s very exciting for us. Or, for example, oral sex, which is fun for both of us. But I’m sure it’s not interesting in my parent’s opinion, so how can I get help from them?"*

Support from friends and colleagues are also helpful in solving couples’ sexual problems. Sh, 29 years old, an electrical engineer man, referred to his colleagues’ support to address each other’s problems.

*"For example, with my co-worker, there were issues that when we had a sexual problem we talked together. Where did you go, what doctor you went to? What did you do? It is 100% helpful".*

Community support systems such as schools, universities, workplace, health centers, and the official media are passive in sex education because of sexual taboos and synonyms of sexuality education by promoting unrestrained behaviors in the society. The lack of specialized and clinical counseling system for sexual problems of couples was identified as one of the weaknesses of the official health system in Iran.

N, a 28-year-old man and accountant, said pre-marital classes as the only formal education in sexual matters did not have the necessary effects.

*"In pre-marital classes at the health center, which in my opinion do not teach a lot, because of a number of limitations".*

His wife (A, 28 years old and a nurse) had a similar view:

*"Sex education for couples in official media and health centers is only about preventing unwanted pregnancies and preventing sexually transmitted diseases. I think there should be valid counseling centers that provide comprehensive information to couples in health centers. So, couples can use this service at a low cost; because the cost of sexual counseling in the private sector is high and most couples cannot afford for this fee".*

### Behavioral skills

The behavioral skills construct in the IMB model is an essential determinant that includes objective skills of individuals to improve sexual life and self-efficacy or believe in their ability to implement sexual enhancement behaviors.

The dominant behavioral skills which were expressed by most couples include applying methods to increase intimacy in marital life as creating a sincere environment, expressing love physically like caressing, kissing, hugging. M, 26 years old, diploma and industrial worker man, describes his sexual skills as such:

*"I try to do what my wife likes and draw her attention to me. Everyone knows what his wife’s disadvantage is, how you can attract her to yourself. When she is sad, I try to get her heart by talking, a surprise, a gift, a flower bunch or touching, kissing and hugging".*

In contrast, his wife (E, 21 years old, art student) refers to the use of her elegance and femininity skills. Like most Iranian women who do not directly ask their husbands for sex, she refers to the exchange of nonverbal messages between her and her husband using femininity skills.

*"Sometimes before my husband comes home, I cook his favorite food, decorate the salad, wear a pretty and seductive dress, makeup, I use a good perfume, or, for example, I would play the song that we listened to at the time of the engagement, sometimes dance, my husband who comes home, finds out that we should do something tonight. He cannot withstand despite being fatigued".*

Sexual interactions without learning communication skills will not be a complete experience. Participating couples referred to verbal and written communication skills such as talking about sexual thoughts, beliefs, feelings, tendencies, and expectations, as well as happy communication such as posting jokes, romance messages, defining and praising of the spouse that leads to increased forgiveness, compatibility, cooperation, commitment, support, and intimacy in their marital life. S, 27-year-old woman, industrial engineer, who has been married for 4 years said:

*"We had a lot of problems early in our lives. We are now forgiving the mistakes of each other, rather than disturbing each other; we share our wishes together and solve our problems by speaking. We forgive each other most of the time. In the past four years we have come to the conclusion that we can’t change each other, so whenever I’m upset, my husband jokes with me, trying to make me laugh and mollycoddle me to make me feel good".*

#### Differences of generations in sexual behaviors

Sexual behavior varies between different ages and cultural, social, and economic groups. In recent years, some social traditions have changed in the Iranian society. Factors like westernization and modernity, access to communication technology, and social media played an important role in these cultural changes. So, the predominant pattern of sexual behaviors among Iranian young people has faced serious change. On the other hand, the rapid technological advancement has led to newer and more diverse demands of individuals, and welfare, freedom, tranquility, and pleasure have become more important. M, 29 years old, bachelor of chemistry and a self-employed man, pointed to the differences in generations in accepted patterns of sexual behaviors.

*"Women need to learn about up-to-date sex, to have sexual relations in accordance with the sexual needs that are currently being promoted in the world. Sexual relationships in our era differ from the sex of my grandparents. They must understand this difference".*

In the same vein, his wife (Z, 26-year-old woman and English translator) compared the sexual behavior of the new generation with their parent’ behaviors.

*"Our mother and father did not like some of sexual behaviors that occurs between husbands and wives nowadays, like the oral relationship or a lot of relationships before having sex (foreplay). They do not like these behaviors and only know intercourse about sex. For example, having anal sex in today’s young people is very common especially among men that I think is the result of seeing porn movies".*

## Discussion

In this qualitative inquiry, we explored the sexual information, motivation, and behavioral skills of newly married couples. The most important finding of the study in the information part was the lack of proper and adequate information for couples at the time of marriage, which led to the formation of erroneous beliefs and attitudes about sexual interactions, especially among women. The couples further stated that correct and timely sexual information that leads to the formation of appropriate sexual attitudes is essential for effective sexual communication. They referred to family and society as the two main institutions in their sexual socialization process. In Anarfi and Owusu’s study [25], family, state, and religion are referred to as three effective institutions in the process of sexual socialization. The dominant approach to sexual issues varies in different cultures, so that some cultures have adopted a clear-cut approach to sexual issues, and in other cultures, these issues are completely concealed, obscure, and even out of reach of individuals. The adoption of each of these approaches in sexual matters goes back to social, cultural, political, and religious interactions [26]. On the other hand, based on the social structuralism model of Berger and Luckmann, introduced by Foucault in the field of sexuality, all sexual matters are defined on the basis of social and cultural standards that reflect the social structures and cultural standards of each society and have a special role in shaping the sexual beliefs and attitudes of people in society [27]. In Iran, as one of the traditional religious communities, the sex-based approach does not set a clear path for the sexuality of individuals, and sexual issues are always in a state of ambiguity, so that Iranians’ sexuality is strongly embedded in the culture of silence, meanings, and traditional scripts, like other contexts [5]. The main reason for the conservative approach of Iranian families that prohibit them from discussing sex education with their children may be their cultural upbringing rather than their religious learning. They are often brought up in a state of ignorance with regards to sexual issues. As a result, they may not be satisfied with their own sexuality or its expression.

For the process of optimal sexual socialization, it is necessary to break the silence of society in the field of sexuality. Given the domination of the strong and rich Islamic ideology in Iranian society and the potential role of religion in the individual and social life of individuals, parents and social institutions can take advantage of the Islamic principles of sexual education and sexual socialization because Islam has emphasized healthy sexual relationships [28]. From the viewpoint of the Quran and religious doctrines, it is not permissible to reject the sexual instinct. Islam does not consider sexual desire to be ugly; instead, it has taken great measures to create deep love and intimacy between the husband and wife in order to establish a solid relationship. God says in the “Quran” (Surah Al Rom, verse 21): “And of His signs is that He created for you spouses from among yourselves, that you may find tranquility in them, and He sets affection and mercy between you. In that, are indeed signs for people who reflect” [29]. However, there are different interpretations of the verses and traditions of Islam in different societies, and Iran is no exception to this rule.

In the dimension of motivation, the most important motive of participating couples in a sexual relationship was paying attention to the sexual need of each other and responding to it through love and mutual interest. Islamic teachings also emphasize on the mutual and unconditional sexual relationship between husband and wife [30]. In a study conducted by Ghorashi et al., the main sexual motivation of participating women for engaging in a sexual relationship was to satisfy men [31]. In the present study, attention to sexual need is mutual and bilateral, which may be due to differences in participants’ characteristics, as in this study, both couples participated but in Ghorashi’s study, only women were present.

Sexual submission is considered as a religious duty for women per the marriage contract. In Islamic cultures, androcentric or patriarchal concepts strongly influence women’s sexuality. Women are culturally conditioned to properly satisfy their husbands and perform their duty as a wife [31, 32]. Females in Khoei et al.’s study considered sexual obedience as the best way to achieve modesty and respect in marital life [32].

In the behavioral skills part, couples pointed to effective communication skills in improving sexual and marital life. In fact, communication skills are the exchange of thoughts, ideas, and needs among couples that help them achieve interpersonal goals [33, 34]. Communication skills are also a set of abilities which provide context for compatibility and positive and useful behavior and enable the person to behave in a decent manner [35]. The lack of direct request of Iranian women for sex from their husbands does not mean that they are inefficient in sexual function, but in contrast, these women are strong sexual beings that have the ability to control and even suppress their sexual desire and use it at the proper opportunity. Also, these women have an adequate self-efficacy to express their sexual request indirectly and to engage their husbands for sexual function by using femininity and creating sexual and appealing attraction.

## Conclusion and recommendation

Our findings support the utility of IMB as a potential basis for understanding married couples’ sexual life. The participants highlighted their need for information related to relevant sexual information and the services provided by the various agents in their living contexts. The couples shared a good comprehension of each other’s means of sexual behaviors. A dominance of religious discourse, non-verbal, mostly physical means of communication was employed by the couples to express or initiate sexual interactions. Our data highlight an implication to expand the motivation structure of the IMB model to incorporate an individual’s sexual understandings and the sexual needs to promote mutual and pleasurable sexual life within the context of Islamic values. Provision of appropriate sexual information and services is recommended for the promotion of individuals’ sexual health as a human right and a medical, evolutionary, social, and religious priority.

## Data Availability

The data that support the findings of this study are available from Fahimeh Bagherikholenjani. Data are also available from the authors upon reasonable request and with permission of Ethics Committee of Hamadan University of Medical Sciences.

## Abbreviations

IMB: Information-Motivation-Behavioral skills
WHO: World Health Organization
